# Retraction Note: Long non-coding RNA MACC1-AS1 promoted pancreatic carcinoma progression through activation of PAX8/ NOTCH1 signaling pathway

**DOI:** 10.1186/s13046-023-02772-4

**Published:** 2023-07-25

**Authors:** Chen Qi, Chen Xiaofeng, Li Dongen, Yang Liang, Xu Liping, Hu Yue, Jiang Jianshuai

**Affiliations:** Department of Hepatobiliary & Pancreatic Surgery, Ningbo First Hospital, No. 59 Liuting Street, Haishu District, Ningbo, 315000 Zhejiang Province China

**Retraction Note:** ***J Exp Clin Cancer Res*** **38, 344 (2019)**

10.1186/s13046-019-1332-7

The Editor-in-Chief has retracted this article. After publication, concerns were raised regarding highly similar features in the published images. Specifically:

• Fig. 2f Invasion si-NC and MACC1-AS1 images appear to contain some highly similar areas;

• Fig. 2f Invasion si-MACC1-AS1 and pWPXL images appear to contain an overlapping area;

• Fig. 5a appears to contain some duplicated patterns within each image.

Additionally, Fig. 3a appears highly similar to Fig. 3e in [1], and Fig. 6b β-actin blots appear highly similar to Fig. 2a GAPDH in [2]

Further checks by the publisher have also found that there appear to be duplicated western blot bands and background features in Fig. 3a, signs of erased bands in Fig. 3e, and background irregularities in Fig. 6b and c. In addition, a number of images in Fig. 2f appear highly similar to those in Fig. S1a and c in [2]; Fig. 2g top images appear highly similar to Fig. S2a in [2], and the bottom images share highly similar features with Fig. S2b in [2].

The Editor-in-Chief therefore no longer has confidence in the presented data.

Jiang Jianshuai has stated on behalf of all co-authors that they agree to this retraction.
